# Using activation status of signaling pathways as mechanism-based biomarkers to predict drug sensitivity

**DOI:** 10.1038/srep18494

**Published:** 2015-12-18

**Authors:** Alicia Amadoz, Patricia Sebastian-Leon, Enrique Vidal, Francisco Salavert, Joaquin Dopazo

**Affiliations:** 1 Computational Genomics Department, Centro de Investigación Príncipe Felipe (CIPF), Valencia, Spain; 2 Bioinformatics of Rare Diseases (BIER), CIBER de Enfermedades Raras (CIBERER), Valencia, Spain; 3 Functional Genomics Node, (INB) at CIPF, Valencia, Spain

## Abstract

Many complex traits, as drug response, are associated with changes in biological pathways rather than being caused by single gene alterations. Here, a predictive framework is presented in which gene expression data are recoded into activity statuses of signal transduction circuits (sub-pathways within signaling pathways that connect receptor proteins to final effector proteins that trigger cell actions). Such activity values are used as features by a prediction algorithm which can efficiently predict a continuous variable such as the IC_50_ value. The main advantage of this prediction method is that the features selected by the predictor, the signaling circuits, are themselves rich-informative, mechanism-based biomarkers which provide insight into or drug molecular mechanisms of action (MoA).

Predicting the best treatment strategy from genomic information is a core goal of precision medicine. In particular, the ability to predict drug response is crucial to establish proper dosages and identify individuals at increased risk for adverse effects^1^. Different attempts to create predictive models of drug response produced gene expression signatures for different aspects of the effect of the drug over the cell culture^2^,^3^,^4^,^5^. Similar attempts were made for responses to chemical compounds^6^,^7^,^8^. However, the prediction accuracy of biomarkers such as gene expression signatures has been questioned in numerous occasions because of their low reproducibility across different studies^9^ and their lack of robustness when challenged with different samples^10^,^11^. Apart from technical or methodological problems, the main difficulty in predicting complex traits, such as drug response, come from the fact that they cannot be understood using methods that ignore the complex gene interactions that may drive cellular response^12^,^13^. Therefore, the knowledge of the underlying molecular mechanism of action (MoA) by which the drug affects cell functionality constitutes a critical step in the prediction of drug responses^14^. With this purpose, several authors have tried to combine biomarkers over biological modules related to cell functionality, such as protein networks^15^,^16^ or biological pathways^17^. Particularly relevant in drug response are signaling pathways, which provide a formal representation of the processes by which the cell triggers specific functional activities in the cell in response to particular stimulus through different circuits of intermediate gene products. Interestingly, the activity of these signaling circuits can directly be related to cell functionalities. Different repositories, such as KEGG^18^, Reactome^19^ and others, contain abundant and detailed information about signaling pathways.

Conventional methods (pathway topology PT-based algorithms^20^) use the topological relationships between the proteins within a pathway to compute a score related to its global activation status from gene expression values^21^,^22^,^23^,^24^ or from mutations^25^. Seeking for a more detailed description of pathway activity, more sophisticated approaches aimed to discover any type of activated sub-network within pathways^26^,^27^,^28^. In particular, more recent methods specifically focus on the estimation of the activity of those sub-networks that can directly be related to cell activity: the signaling circuits that receive a stimulus and trigger a response^29^,^30^,^31^. Preferably, the activity of such stimulus-response signaling circuit could be inferred from (phospho)proteomic and chemoproteomic experiments^32^. However, the production of these types of data still results relatively complex and consequently very few datasets are available. Nevertheless, abundant gene expression data on comparative studies of drugs^33^,^34^,^35^,^36^ and other compounds^37^,^38^,^39^ are available, constituting an invaluable resource for comparative studies of drugs and cell lines^14^. The estimation of the activity of signaling circuits from gene expression data^29^,^30^,^31^ provide a rich-informative type of biomarkers, thereinafter called mechanism-based biomarkers, whose direct relationship to cell functionalities can be used to suggest mechanistic explanations for the molecular basis of complex traits^40^.

Here we present a simple way of recoding gene expression values into mechanism-based biomarkers. Such biomarkers are subsequently used in an innovative way in the context of prediction. In particular, the method presented here is applied to predict a complex trait like drug sensitivity. Two large-scale datasets in which different cell lines are treated with a variety of drugs^35^,^36^ are used to illustrate how the predictions based on the proposed mechanism-based biomarkers are not only accurate but also provide relevant clues to understand the MoA of the drugs assayed.

Additionally, a freely available web tool for carrying out the predictions has been developed.

## Results

### Use of signaling circuit activation probabilities in the context of prediction

Gene expression values are recoded into signaling circuit activities (elementary components of signaling pathways) using a probabilistic model^30^ as described in Methods. These activity values are further used as mechanism-based biomarker features for prediction purposes. Typically predictors are built by first selecting the best informative features (mechanism-based biomarkers here) and then applying a prediction algorithm.

For each dataset, microarray gene expression data were normalized with RMA^41^. Normalized gene expression values were transformed into probabilities of signaling circuit activation^30^. Such probabilities are considered mechanism-based biomarkers and are the features used in the proposed predictive framework. Circuits whose activation statuses do not change across the conditions studied (invariant biomarkers) were initially discarded. Then, feature selection is carried out over the remaining circuits. Here, we have used Correlation-based Feature Selection (CFS). The selected biomarkers are subsequently used by the predictor algorithm. Here, we have used a regression based on Support Vector Machine (SVM)^42^ (SVM ɛ-regression, as implemented in the e1071 R library^43^) to predict the value of a continuous variable such as the IC_50_. Strictly speaking, in the case of SVM the previous step of most informative variables could be skipped.

The accuracy of the classification obtained was evaluated by ten-fold cross validation^44^, using the following parameters: total mean square error and squared correlation coefficient.

### Prediction of IC_50_ values for cancer drugs using circuit activity values as features

Two gene expression datasets of human tumor cell lines screened for different drugs with the corresponding IC_50_ measurements available were used. One of them, the CGP (ArrayExpress ID: E-MTAB-783)^35^ was used to train the predictor and the other one, CCLE (GEO ID: GSE36139)^36^, to validate the predictions. Common data from both datasets were selected, resulting in a total of 317 cell lines, 12 cancers and 7 drugs. CGP data were normalized with RMA^41^ and normalized gene expression values were transformed into probabilities of signaling circuit activation^30^. Finally, a predictive model was obtained for the CGP data with a SVM ɛ-regression as explained in Methods section.

**Figure 1:**
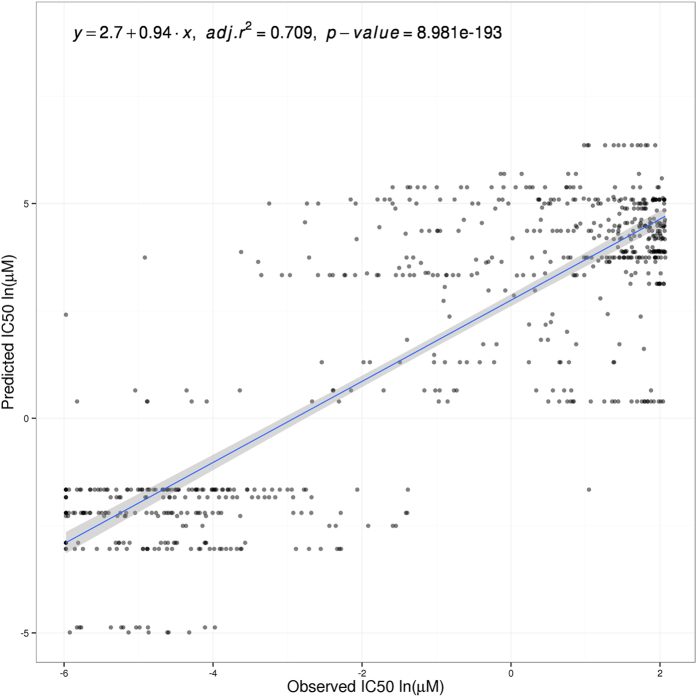
Predicted versus actual CCLE IC_50_ values using signaling circuit activities as features. Values are represented per cell line and compound (418 cell lines, 7 compounds and 6 cancers). Data values are in natural logarithm of micro-Molar units. Linear regression is specified with intercept and slope values. Adjusted R-squared and p-value are also included.

The predictor model obtained with CGP data is used to predict IC_50_ values in the CCLE dataset. Gene expression values were normalized as explained in Methods and used to calculate signaling circuit activation probabilities. Then, these values were used to predict an IC_50_ value for each cell line and drug. Figure 1 shows the agreement between predicted a real values. There is a highly significant positive correlation (r = 0.709, p = 8.98 × 10^−193^) between the expected, real IC_50_ values measured in the CCLE dataset and the values predicted by regression, based on the CGP dataset, which clearly confirms the validity of the prediction framework proposed here.

**Figure 2:**
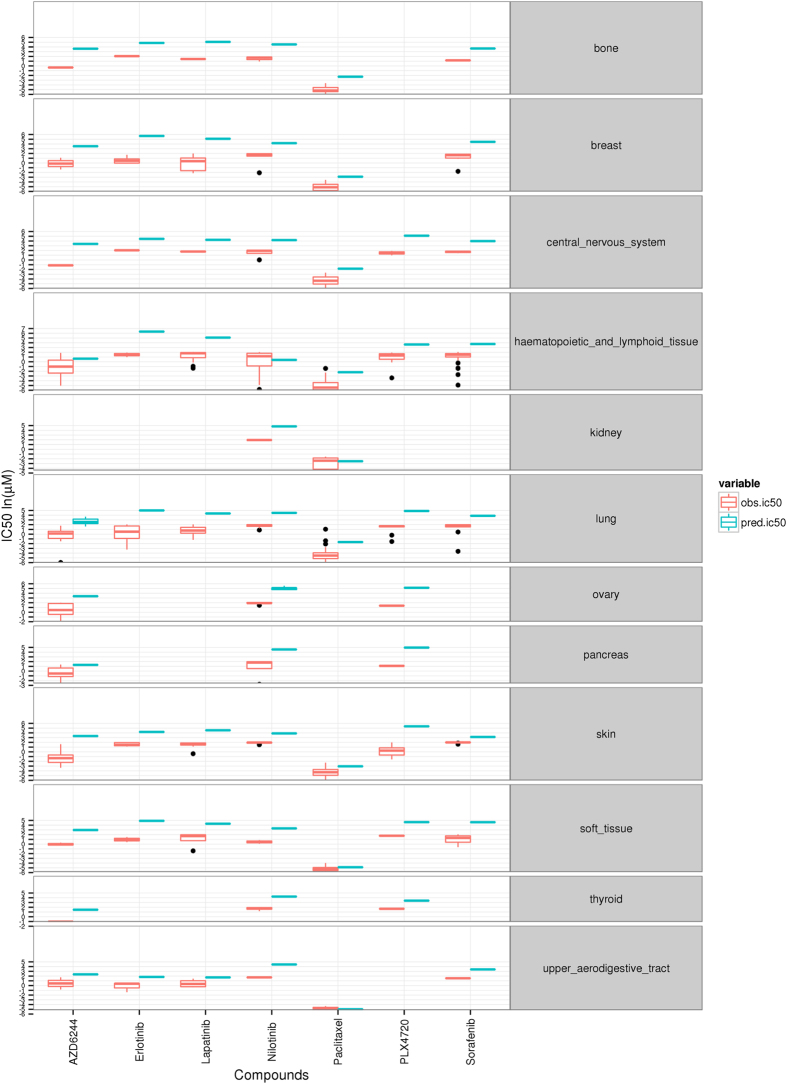
CCLE predicted and observed IC_50_ values per cancer and drug.

Figure 2 shows the predicted IC_50_ values and the corresponding real IC_50_ values available for the CCLE dataset averaged by tissue. Both predicted and real IC_50_ values were compared by estimating the root mean square error (RMSE) (Table 1). While there are some discrepancies, a global RMSE of 3.31, including all cancers and drugs, demonstrates a quite reasonable accuracy for the prediction. Specific cancers and drugs for which the prediction is especially good are: upper aerodigestive tract (RMSE = 0.52) and soft tissue (RMSE = 0.64) with Paclitaxel. The most extreme discrepancies occur in breast for Erlotinib (RMSE = 5.19) and Lapatinib (RMSE = 5.28). In general, breast tissue shows a poorer prediction than the rest of tissues. It could be due to the fact that some key pathways, such as the ERBB signaling pathway, are under-represented among the features chosen for the prediction. It can also be due to the fact that RAS signaling pathway, relevant in breast cancer, was not modeled here. Also, in skin cancer cell lines, PLX4720 (RMSE = 5.3) AZD6244 (RMSE = 4.71) seem to have selected different features to the rest of drugs which perform better in the classification (RSMEs between 1.19 and 3.22). The use of SVM, which used a combination of features for the prediction, makes difficult finding a unique explanation for the discrepancies.

### Comparison to predictions of IC_50_ values for cancer drugs derived when all the gene expression values are used as features

**Figure 3:**
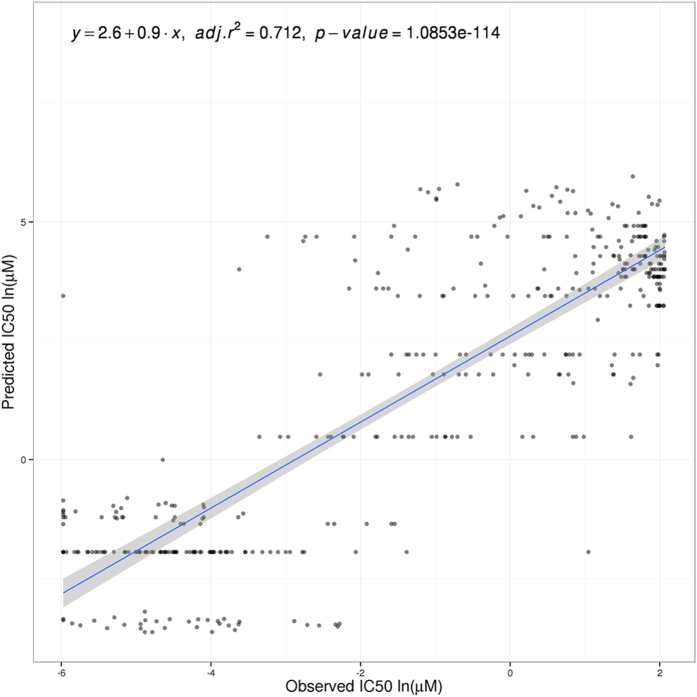
Predicted versus actual CCLE IC50 values using normalized gene expression values as features. Values are represented per cell line and compound (418 cell lines, 7 compounds and 6 cancers). Data values are in natural logarithm of micro-Molar units. Linear regression is specified with intercept and slope values. Adjusted R-squared and p-value are also included.

The potential of the full set of gene expression values as features for predicting drug sensitivity has already been proven in a recent study^45^. Here we have trained the predictor used in this study using directly the normalized gene expression values (instead of transforming them into signaling activities) as features. Figure 3 plots the predicted IC_50_ values and the corresponding real IC_50_ values for the CCLE dataset. The predictive power of all the genes also resulted in a highly significant positive correlation (r = 0.712, p = 1.09 × 10^−114^) between the expected, real IC50 values measured in the CCLE dataset and the values, predicted by regression, based on the CGP dataset.

It must be taken into account that signaling circuit features, as defined here, will only account for the effect of the condition studied over the modeled signaling pathways. Information on any effect unrelated to signaling or related to yet unknown or undescribed signaling pathways is missing in the proposed method. In spite of this fact, the direct or indirect impact of the drugs studied on the signaling circuits is enough to produce quite reliable predictions (Fig. 1) of similar precision than the global gene expression, which potentially captures the whole reaction of the cell to the conditions studied.

### MoA suggested by the mechanism-based biomarkers selected

**Figure 4:**
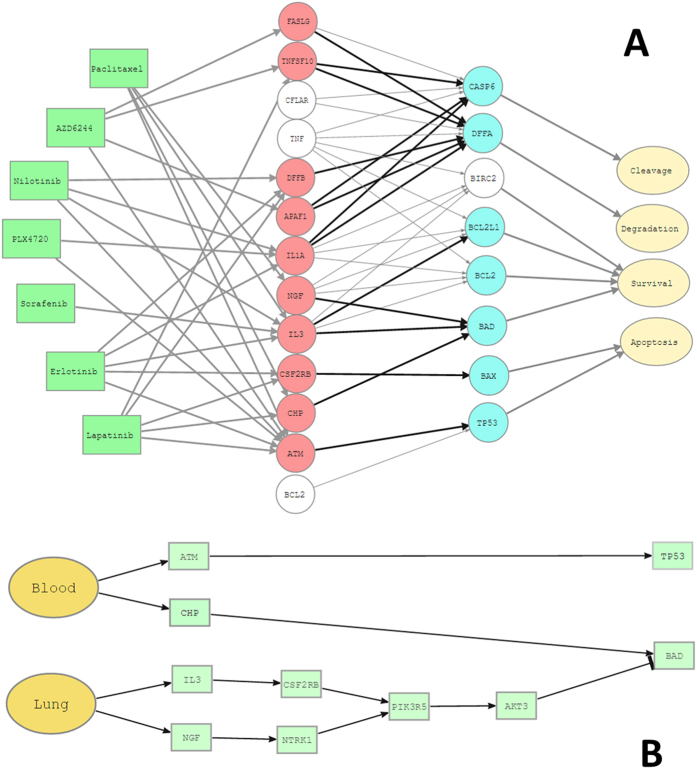
Possible mechanism of action (MoA) of the different drugs in the apoptosis pathway as suggested by the signaling circuits selected by the predictor. (**A**) Drugs (left in green) that act over the different circuits defined by the receptor (in red) and effector (in blue) proteins, respectively. Cell functionalities triggered by the circuits are labeled in pale yellow. The circuits not affected by any of the drugs are dimmed in gray. (**B**) Specific circuits over which the Paclitaxel drug acts in both Blood and Lung cancer cell lines. The circuits affected trigger survival and apoptosis.

The proposed methodology selects the most predictive signaling circuits within each pathway modeled for each drug analyzed in each tissue tested. Supplementary Fig. S1 displays the signaling circuits selected for each drug/tissue combination. An interesting observation is the pervasiveness with which drugs affect to pathway activity. Almost all the pathways are affected in at least one circuit by any of the drugs in at least one of the tissues tested. There are, however, a few exceptions, such as the JAK-STAT signaling pathway, which is never affected by AZD6244 or Sorafenib; or NOTCH signaling pathway which is never affected by Nilotinib or AZD6244, along with a few more examples. The signaling circuits selected are those that exhibit the most dramatic change in activity among all the circuits affected by the drug in a particular tissue. Table 2 shows the bibliographic references that report the alteration of pathways by the studied drugs. There are numerous reports for some pathways, which are extensively affected by all the drugs, as the Apoptosis pathways, affected by Paclitaxel^46^, AZD6244^47^, Nilotinib^48^, PLX4720^49^, Sorafenib^50^, and Lapatinib^51^. Actually, it is interesting to see how different drugs affect the apoptosis pathway in different ways. For example, Sorafenib is the only drug that affects the pathway by exclusively inhibiting survival. It has been documented that this drug induces apoptosis by down-regulating the anti-apoptotic protein Mcl-1 via transcriptional inhibition and protein degradation^50^. Actually, the Mcl-1 is in the PI3K-ATK pathway and triggers survival in the Apoptosis pathway. Supplementary Fig. S1 shows how survival is inhibited by two circuits ending, respectively, in proteins BCL2 and BCL2L1. For the rest of drugs, however, the most common mechanism is the activation of the apoptosis via TP53 protein, along with a number of complementary circuits that activate functions complementary to apoptosis, such as degradation or cleavage of caspase substrate. Figure 4A shows the different signaling circuits used by the different drugs to cause cell death, which illustrate the diversity of drug MoAs, which can also differ across cell lines (Supplementary Fig. S1).

There are drugs whose effect on different pathways have extensively been documented in the literature and confirm the results here presented. For example, Paclitaxel is known to affect to the Apoptosis pathway^46^ (see Fig. 4B), the Insulin signaling pathway^52^, the WNT signaling pathway^53^, the Calcium signaling pathway^54^, the Hedgehog signaling pathway^55^, the JAK-STAT signaling pathway^56^, the p53 signaling pathway^57^, the Chemokine signaling pathway^58^, the PPAR signaling pathway^59^, the Toll-like receptor signaling pathway^56^ and the VEGF signaling pathway^60^. Supplementary Table S1 presents detailed references that link the drug to the pathway affected in one of the cancers in which the method has selected signaling circuits from it.

Although a detailed analysis of the MoAs of each drug in each cancer would be excessively long and is beyond the scope of this paper, it is clear that the IC_50_ prediction is systematically based on a subset of features which have a mechanistic relevance in the action of the drug.

### Experimental validation of relevant activity changes predicted

The schema used in this study encompasses the use of a training set (CGP) in which the predictors have been derived and another independent dataset (CCLE) in which the accuracy of the predictors have been demonstrated. Such demonstration involves the observation of differential activity in the signaling status of the selected circuits in the independent dataset and can be considered *per se* an experimental validation of the existence of such changes. However, the unlikely possibility that some of these circuits were artefactual cannot be completely ruled out because, although all the genes involved in them were actively being transcribed, they could be not translated to proteins or these could be inactive.

Although phospho-proteomic data are scarce, there is a dataset from a study of global phosphorylation changes upon Erlotinib treatment of lung adenocarcinoma cell lines^61^, which can be used to confirm the existence of proteins involved in the selected circuits and the change of their activation status. The study contains data on H3255, a lung adenocarcinoma cell line sensitive to EGFR-directed tyrosine kinase inhibitors, and H1975, another lung adenocarcinoma cell line resistant to first-generation reversible EGFR-TKIs, such as Erlotinib. Both cell lines are included in the CGP dataset used to generate the prediction models. Erlotinib in lung cell lines affects to a total of 23 signaling circuits across 15 pathways and include 78 nodes (see Supplementary Fig. S1). Phosphorylation data are not exhaustive and the study contains only ratio values for total of 21 proteins belonging to the circuits selected by the predictor (Table 3).

**Figure 5:**
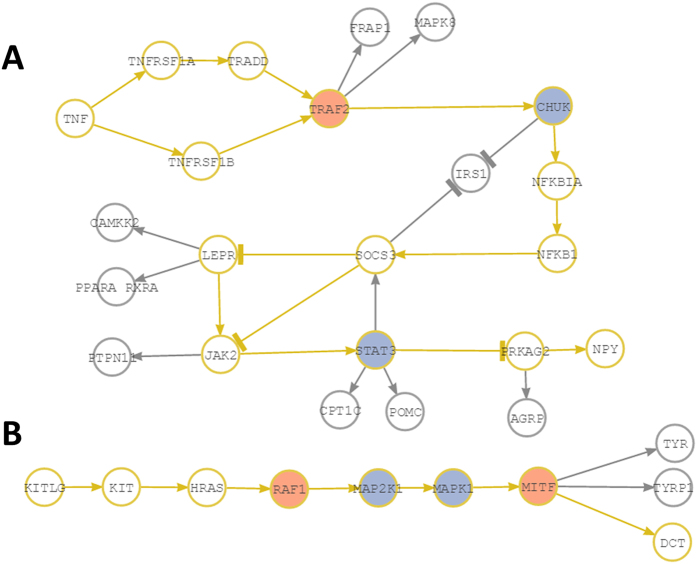
Two examples of circuits selected by the predictor. The circuits are represented in yellow. Arrowheads indicate activations and “T” heads indicate inhibitions. (**A**) Circuit TNF-NPY from the Adipocytokine signaling pathway (hsa04920). (**B**) Circuit SCF(KITLG)-DCT from the Melanogenesis pathway (hsa04916). Nodes in red contain proteins found to be hyperphosphorylated when comparing treated versus untreated lung cancer cells. Nodes in blue contain proteins found to be dephosphorylated in the same comparison.

In general there is a good agreement between the change of phosphorylation status of the proteins measured that belong to a circuit selected by the predictor and the change in signaling obtained upon the application of the Pathiways tool^30^ for this circuit. Some apparent contradictions can easily be explained by the topology of the circuits. For example, in the case of insulin signaling pathway, *PDPK1* shows a dephosphorylation and *AKT1* shows inconclusive results (hyper-phosphorylation in one cell line and dephosphorylation in the other one) although Pathiways predicts an increase in signaling. However, *aPKC*, at the end of the circuit (according KEGG) can also be directly activated by *INPP5* (*SKIP* node in KEGG), which explains the apparent discrepancy in the results. Another example is the circuit *TNF*-*NPY* in the Adipocytokine signaling pathway (Fig. 5A), which is predicted to be activated by Pathiways. The observed phosphorylation pattern is complex and involves hyper-phosphorylations and dephosphorylation. However, essentially what happens is that TRAF2 is activated and transmit the signal to *CHUK* node, where one of the three proteins (*IKBKB*) present in the node is dephosphorylated. There is no data on successive steps of signal transmission until the *STAT* protein, which is dephosphorylated. *STAT* is an inhibitor of *PRKAG2* that transmit the last step of signaling to *NPY*, which allows concluding that the signal ultimately arrives to *NPY*, as predicted by Pathiways for this circuit selected by the predictor. Another interesting case is the circuit *SCF*(*KITLG*)-*DCT* from the Melanogenesis pathway (Fig. 5B). This is a lineal circuit with no bifurcations. After the hyperphosphorylation of the protein *RAF1*, the unique component of the node, there are two nodes that contain dephosphorylated proteins in the circuit (*MAP2K1* and *MAPK1*). However, it seems that other proteins in the nodes managed to transmit the signal, because finally, *MITF*, the ultimate responsible of transmit the signal to *DTC* is hyperphosphorylated again. This agrees again with the Pathiways prediction of increase in the signal transmission in this circuit.

### Software for the derivation of mechanism-based predictors

A web page which allows transforming gene expression values into signaling circuit activities (mechanism-based biomarkers) and further using them to calculate the best predictor of the studied experimental conditions is available at: http://pathiways.babelomics.org/.

The program inputs “.CEL” files. Gene expression values are transformed into circuit activation probabilities^30^. These probabilities are used to derive a predictor of the conditions studied. Additionally, a matrix of samples x probabilities can be saved to be subsequently used in other programs which can derive predictors by other algorithms (for example in Babelomics^62^), if desired. The program also provides estimations of the differentially activated circuits as described elsewhere^30^.

## Discussion

Due to the inherent complexity of the cell, phenotypes cannot be understood as the result of the action of only one or a few genes^12^,^13^. Consequently, conventional approaches to study the phenotype must evolve from a gene-centric perspective towards a systems-biology-oriented view that considers the combined action of several functionally related genes^63^,^64^,^65^. Therefore, new approaches that transform individual gene measurements (e.g. gene expression levels) into parameters that describe functional activities of biological modules open the way to the definition of a new type of mechanism-based biomarkers which can be used to predict complex phenotypes such as disease status or drug responses^66^.

In particular, the identification and knowledge of drug MoAs is crucial in pharmacogenetic studies^67^. The fact that many drugs target signal transduction processes requires of a detailed understanding of the MoA at the signaling level, not only in the specific tissue in which the drug is aimed to act but also in other tissues that may suffer off-target effects^14^. Understanding such mechanisms could have an enormous impact in many aspects of drug development and personalized therapies^68^.

The prediction strategy proposed here introduces the use of mechanism-based biomarkers (signaling circuits), responsible for specific cell functionalities, whose anomalous activity could be the ultimate cause of a phenotype. Such biomarkers throw light on possible drug MoAs^40^,^69^, given that changes in the activity of the individual molecules is understood within the context of the system conformed by the signaling circuits^64^,^65^.

The predictions obtained are quite reasonable and even slightly better than the predictions obtained using all the genes, in agreement with previous observations^70^. It must be taken into account that: 1) the proposed mechanism-based biomarkers capture only the part of the effect of the drugs that either directly or indirectly affect cell signaling and 2) the predictions obtained with all the genes use much more variables (over 20,000 genes) than the corresponding ones obtained here with the signaling circuits (using only about 800 genes involved in the circuits).

Moreover, the specific circuits selected by the predictor, in the cases in which a validation has been possible using phosphorylation data, are not only transcriptionally active but also the proteins have been produced and the corresponding changes in their phosphorylation statuses are observed. Although gene expression does not necessarily imply its translation and its subsequent activation, our results strongly suggest that, when collective gene up-regulation (or down-regulation) occurs within the context of a pathway, it can be considered a reliable proxy of activation (or deactivation) of the corresponding signaling circuits.

Therefore, the use of mechanism-based biomarkers in a prediction context not only provides mechanistic explanations on the phenotypes studied but, in addition, it seems to produce comparatively better predictions.

The proposed methodology can be easily used through the software provided. Here, the methodology was focused on gene expression data obtained from microarrays because of its availability. Gene expression values can be obtained by other methodologies, such as RNA-seq^71^, providing the data compared are in the same scale (this is the objective of the normalization process).

## Methods

### Data sources

Two published large-scale datasets from the cancer genome project (CGP) (E-MTAB-783 in the ArrayExpress repository)^35^ and the cancer cell line encyclopedia (CCLE) (GSE36139 accession number in the Gene Expression Omnibus repository)^36^ were used in this study. Both datasets provide gene expression information of human tumor cell lines which have been screened for different drugs and the concentration at which the drug response reached an absolute inhibition of 50% (IC50). CGP dataset provides information about 138 drugs, 661 cell lines (including NCI-60 cell lines) which correspond with 17 cancers; on other hand, CCLE dataset includes information about 24 drugs, 493 cell lines which correspond with 24 cancers. Common data from both datasets were filtered for a total of 355 cell lines, 12 cancers and 7 drugs.

Phospho-proteomic data were obtained from a study of global phosphorylation changes upon Erlotinib treatment of lung adenocarcinoma cell lines^61^. Supplementary material of this study includes information of EGF stimulation (stimulated/untreated cells) and Erlotinib inhibition (treated/untreated cells) SILAC ratios.

### Data preprocessing

Microarray normalization was carried out using the function *RMA* in the *affy* library^41^ of Bioconductor^72^, as implemented in Babelomics platform^62^.

For the phosphorylation data, mean ratios gene protein were computed using its phosphosites’ phosphorylation ratios and a ratio cutoff was applied to define hyperphosphorylation (hyperP) and dephosphorylation (deP) states. The ratio cutoff was defined as follows: > 1.1 increased, 0.9–1.1 unchanged and < 0.9 decreased. Then, hyperphosphorylation (hyperP) was defined as having a decreased ratio of stimulated/control and an increased ratio of treated/stimulated, and dephosphorylation (deP) when having an increased ratio of stimulated/control and a decreased ratio of treated/stimulated.

### Derivation of mechanism-based biomarkers (signaling circuit activation probabilities) from gene expression values

**Figure 6:**
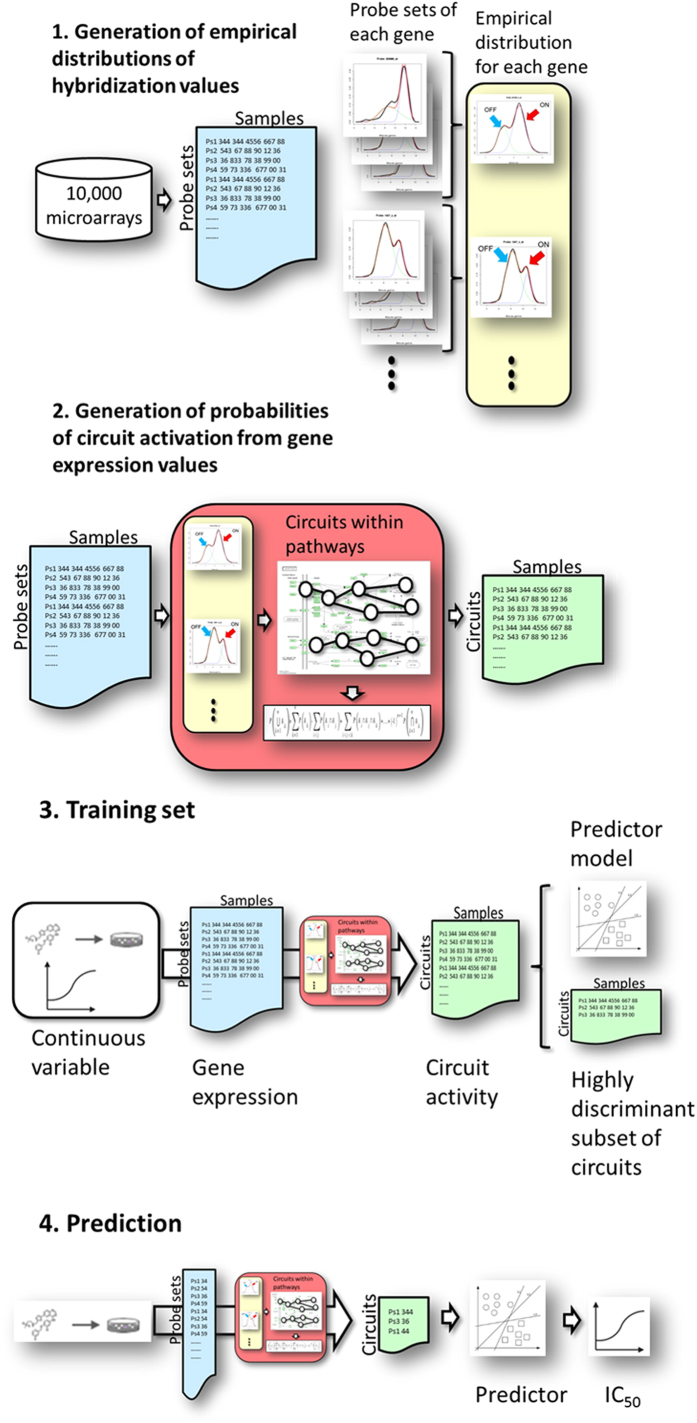
Steps for the generation and use of predictors based on signaling circuit activities. Step 1: generation of the empirical distribution of probeset values. A collection of more than 10,000 microarrays representing an enormous diversity of conditions is collected from the GEO database. For any of the probesets, an empirical distribution is derived and a mixture model is used to define the highest value peak, which corresponds to an active probe (ON), and the lowest peak that correspond to the probeset (OFF). Gene values can be obtained by summarizing the corresponding probeset values. Step 2: Given one or several microarrays, the probeset values can be contrasted with the empirical distribution values to obtain the corresponding activity probabilities which are used to derive gene activity probabilities. These, within the context of the circuits defined, are used to estimate circuit activity probabilities. Step 3: an initial training set is required to derive obtain the predictor. Gene expression values from individuals from two classes, or from different treatments (dosage, time, etc.) are obtained and transformed (as described in step 2) into the corresponding profiles of signaling activities. Then, a feature selection method obtain a sub list of highly discriminative circuits which is used to train the predictor (see below). Step 4: once the predictor is trained it can be used to predict class membership for an unknown sample (or to predict a continuous value from gene expression measurements). Gene expression values from the sample are transformed into the corresponding pattern of signaling circuit activities (see step 2) of the sample. The predictor is then used to predict the class to which most likely the sample belongs to. Identically, gene expression values of a series of conditions can be used to predict the corresponding continuous value (not shown in the figure).

Signaling circuit activation probabilities are inferred from the estimations of gene activation probabilities corresponding to the proteins involved in the circuit. Briefly, a mixture distribution is used on a large dataset of reference microarrays to derive empirical distributions of expression values corresponding to activated and deactivated states of the probe sets in the microarrays. Probesets can be used to summarize gene expression values (Fig. 6, step 1). Within this analytic framework, gene expression is taken as a proxy of protein expression and, consequently, protein activity^26^,^27^,^30^,^31^,^73^.

The step 2 in Fig. 6 illustrates how a matrix of probeset expression values can be converted into a matrix of signaling circuit activation probabilities. Empirical distributions for probe sets previously derived are used to assign a probability of activation to probe sets in the studied microarrays^30^,^31^,^73^. Then, probeset activation values are combined to derive probabilities of gene activation.

On the other hand, signaling circuits are defined as the sub-pathways (within pathways taken from KEGG database^18^) that transmit signals from a receptor node to an effector node. Such circuits can have bi- or multi-furcations and typically consist of nodes that activate other nodes but they can also contain nodes that inhibit the activity of other nodes. Such nodes can be composed of one or several proteins. Finally, node activation probabilities are obtained by combining the probability activation values corresponding to all the genes that comprise the node (see details in^30^,^31^). Once probabilities of activation for each node in the circuit have been estimated, the probability of signal transmission can be modeled as a simple probabilistic product using the inclusion-exclusion principle^30^,^31^ (red box in the step 2 of Fig. 6 summarizes this procedure).

Probabilistic models were obtained as described above for the signaling circuits defined within a total of 26 KEGG pathways for *Homo sapiens* and 18 for *Mus musculus*. These correspond to the general categories Environmental Information Processing and Cellular Processes, which include important processes and systems such as Signal Transduction (ERBB, WNT, NOTCH, JAK-STAT, calcium, VEGF, HEDGEHOG and mTOR signaling pathways), Signaling Molecules and Interaction (neuroactive ligand-receptor interaction, cell adhesion molecules, cytokine-cytokine receptor interaction and EMC-receptor interaction), Cell Growth and Death (apoptosis and p53 signaling pathway), Cell Communication (GAP junction and tight junction), Endocrine System (insulin signaling pathway, adipocytokine signaling pathway, PPAR signaling pathway, GnRH signaling pathway and melanogenesis) and Immune System (toll-like receptor signaling pathway, B cell receptor signaling pathway, T cell receptor signaling pathway, Fc epsilon RI signaling pathway, antigen processing and presentation, and chemokine signaling pathway).

### Derivation of predictors based on mechanism-based biomarkers

Prediction methods require of an initial training set to derive a trained predictor (Fig. 6, step 3) which can be further used to predict the corresponding value of a continuous variable for a new sample (Fig. 6, step 4). The step 3 in Fig. 6 summarizes how the training set is used for the training phase of the predictor. Gene expression values from different treatments are obtained and transformed as described in step 2 (Fig. 6) into the corresponding profiles of signaling activities. Since signaling circuit probabilities are used as mechanism-based biomarkers to predict drug sensitivity values, those circuits showing no variability across the treatments were initially discarded. Then, a sub list of highly discriminative circuits is obtained using Correlation-based Feature Selection (CFS) method^74^ as feature selection algorithm (Fig. 6, end of step 3). Drug sensitivity prediction was carried out using the highly discriminant subset of signaling circuit activities. The prediction algorithm used was Support Vector Machine (SVM)^42^ as implemented in the *e1071* R library^43^. SVM ɛ-regression was performed selecting best γ and cost parameters among different values tested (10, 100 with cost values; 10^−6^, 10^−5^, 10^−4^, 10^−3^ γ values), by optimizing the mean squared error of the model with a 10-fold cross-validation.

In the particular case of the SVM algorithm, the previous feature selection step is not necessary for prediction purposes, unless a subset of relevant features is sought. K-fold cross validation (with K = 10) was used in the training step of the models. The CGP dataset^35^ was used to train the predictor and obtaining the prediction model (Fig. 6, end of step 3).

Once the predictor is trained it can be used to predict a drug sensitivity value from gene expression measurements in a new, unknown sample (step 4 Fig. 6).

Methods for feature selection, classifiers and performance evaluation are implemented in Babelomics platform^62^.

## Additional Information

**How to cite this article**: Amadoz, A. *et al.* Using activation status of signaling pathways as mechanism-based biomarkers to predict drug sensitivity. *Sci. Rep.* **5**, 18494; doi: 10.1038/srep18494 (2015).

## Supplementary Material

Supplementary Information

## Figures and Tables

**Table 1:** RMSE per cancer type and drug in the CCLE dataset.

Cancers	Drug Paclitaxel	AZD6244	Nilotinib	PLX4720	Sorafenib	Erlotinib	Lapatinib
Lung	2.99	3.59	2.77	3.85	2.82	5.08	3.87
Haematopoietic and lymphoid tissue	2.98	2.68	2.42	3.33	3.05	4.88	3.89
Bone	3.96	3.01	NA	2.5	2.78	3.61	3.96
Skin	1.63	4.71	2.02	5.3	1.19	2.66	3.22
Ovary	NA	3.33	3.18	3.77	NA	NA	NA
Central nervous system	2.67	4.51	2.83	3.66	2.29	2.41	2.47
Pancreas	NA	2.08	4.34	3.85	NA	NA	NA
Soft tissue	0.64	3.01	2.88	2.88	3.77	3.99	3.59
Breast	2.23	3.87	3.17	NA	3.67	5.19	5.28
Upper aerodigestive tract	0.52	2.3	2.73	NA	1.86	2.17	1.49
Kidney	1.22	NA	2.88	NA	NA	NA	NA
Thyroid	NA	2.51	2.58	1.71	NA	NA	NA

NAs appear when not enough data were available.

**Table 2:** Pathways known to be affected by different drugs with the corresponding bibliographic citation.

Pathways	Drug Paclitaxel	AZD6244	Nilotinib	PLX4720	Sorafenib	Erlotinib	Lapatinib
Apoptosis pathway	46,55	47	48	49	50		51
Insulin signaling pathway	52	75			76		77
mTOR signaling pathway		78			79	80	81
WNT signaling pathway	53	82		83	84	85	
Adipocytokine signaling pathway						86	86
Calcium signaling pathway	54					87	
ERBB signaling pathway					88	89	89
Hedgehog signaling pathway	55		90		91	92	
JAK-STAT signaling pathway	56					93	
p53 signaling pathway	57				94	95	
Chemokine signaling pathway	58				96		
PPAR signaling pathway	59						
Toll-like receptor signaling pathway	56						
VEGF signaling pathway	60				97		
B cell receptor signaling pathway					98		

**Table 3:** Phosphorylation summary per protein. Mean ratios per gene were computed using its phosphosites’ phosphorylation ratios and a ratio cutoff was applied to define hyperphosphorylation (hyperP) and dephosphorylation (deP) states or no change over the threshold (NC).

Protein	H3255	H1975	Pathway (KEGG ID)	Circuit	Signalling	Node Proteins
ADCY2	deP	deP	Calcium signaling pathway (hsa04020)	CHRM1-ATP2A1	Down	ADCY1 ADCY2 ADCY3 ADCY7 ADCY8 ADCY9 ADCY4
SPECC1L	deP	NA	Calcium signaling pathway (hsa04020)	CHRM1-ATP2A1	Down	CHRM1 CHRM3 CHRM5 ADORA2A SPECC1L ADORA2B ADRB1 ADRB2 ADRB3 DRD1 DRD5 HRH2 HTR4 HTR5A HTR6 HTR7
ATP2A2	NC	NA	Calcium signaling pathway (hsa04020)	CHRM1-ATP2A1	Down	ATP2A1 LOC100510514 ATP2A2 ATP2A3
ADCY9	hyperP	NA	Calcium signaling pathway (hsa04020)	GPCR-SERCA	Down	ADCY1 ADCY2 ADCY3 ADCY7 ADCY8 ADCY9 ADCY4
AKT1	deP	hyperP	Insulin signaling pathway (hsa04910)	SKIP(INPP5K)-aPKC	Up	AKT3 AKT1 AKT2
PDPK1	deP	NA	Insulin signaling pathway (hsa04910)	SKIP(INPP5K)-aPKC	Up	PDPK1
DFFA	hyperP	NA	Apoptosis (hsa04210)	DFFB-DFFA	Down	DFFA
ICAM1	NA	deP	Cell adhesion molecules (CAMs) (hsa04514)	ICAM1,2,3-ITGAL	Down	ICAM1
IKBKB	deP	NA	Adipocytokine signaling pathway (hsa04920)	TNF-NPY	Up	CHUK IKBKB IKBKG
NFKB2	NA	NC	Adipocytokine signaling pathway (hsa04920)	TNF-NPY	Up	NFKB1 NFKB2 RELA
PRKAA1	NC	NA	Adipocytokine signaling pathway (hsa04920)	TNF-NPY	Up	PRKAG2 PRKAG3 PRKAA1 PRKAA2 PRKAB1 PRKAB2 PRKAG1
PRKAA2	NC	NA	Adipocytokine signaling pathway (hsa04920)	TNF-NPY	Up	PRKAG2 PRKAG3 PRKAA1 PRKAA2 PRKAB1 PRKAB2 PRKAG1
STAT3	NA	deP	Adipocytokine signaling pathway (hsa04920)	TNF-NPY	Up	STAT3
TRAF2	hyperP	hyperP	Adipocytokine signaling pathway (hsa04920)	TNF-NPY	Up	TRAF2
MAPK1	deP	deP	Wnt signaling pathway (hsa04310)	Nkd-RhoA	Down	MAPK8 MAPK9 MAPK10
MAPK1	deP	deP	Melanogenesis (hsa04916)	SCF(KITLG)-DCT	Up	MAPK1 MAPK3
MAP2K2	deP	deP	Melanogenesis (hsa04916)	SCF(KITLG)-DCT	Up	MAP2K1 MAP2K2
MAPK3	deP	deP	Melanogenesis (hsa04916)	SCF(KITLG)-DCT	Up	MAPK1 MAPK3
MITF	NA	hyperP	Melanogenesis (hsa04916)	SCF(KITLG)-DCT	Up	MITF
RAF1	hyperP	NA	Melanogenesis (hsa04916)	SCF(KITLG)-DCT	Up	RAF1
TP53	hyperP	hyperP	p53 signaling pathway (hsa04115)	MDMX-Wip1(PPM1D)	Down	TP53

NCs indicate that the protein exist although the phosphorylation status has nos significantly changed. NAs correspond to conditions for which no data are available. For easier identification, circuits have been named according the names of the input and output nodes as they appear in KEGG. The column labelled as signaling contains indications on the change in signal activity of the circuits as predicted by the Pathiways tool^30^ using transcriptomic data of the untreated cell lung line vs the Erlotinib treated one. The last column contains the proteins present in the node of the protein for which phosphorylation data is available.
