# Supplementary material for: Using activation status of signaling pathways as mechanism-based biomarkers to predict drug sensitivity
Source: Sci Rep. 2015 Dec 18;5:18494. doi: 10.1038/srep18494 (PMC4683444; doi:10.1038/srep18494)
Supplement: Supplementary Information [file srep18494-s1.pdf]

Using activation status of signaling pathways as mechanism-based biomarkers to predict drug sensitivity.

Alicia Amadoz, Patricia Sebastian-Leon, Enrique Vidal, Francisco Salavert, Joaquin Dopazo

Supplementary Figure S1 - Signaling circuits selected by the predictor. Columns represent the different cell lines assayed with the different drugs. Files list signaling circuits (represented by the pair receptor/effector nodes, which generally correspond to only one protein, but sometimes are multiprotein nodes), grouped by pathways. Black squares are those circuits selected in the feature selection process, using CFS and SVM to predict IC50 for each drug/tissue combination. The results were obtained in the in the CGP (ArrayExpress ID: E-MTAB-783) [33] dataset.

Supplementary Table S1 – Pathways in which the predictor (feature selection using CFS and SVM used to predict IC_{50}) selected signaling circuits with high predictive power and bibliographic references that explain how the pathway is affected by the drug.

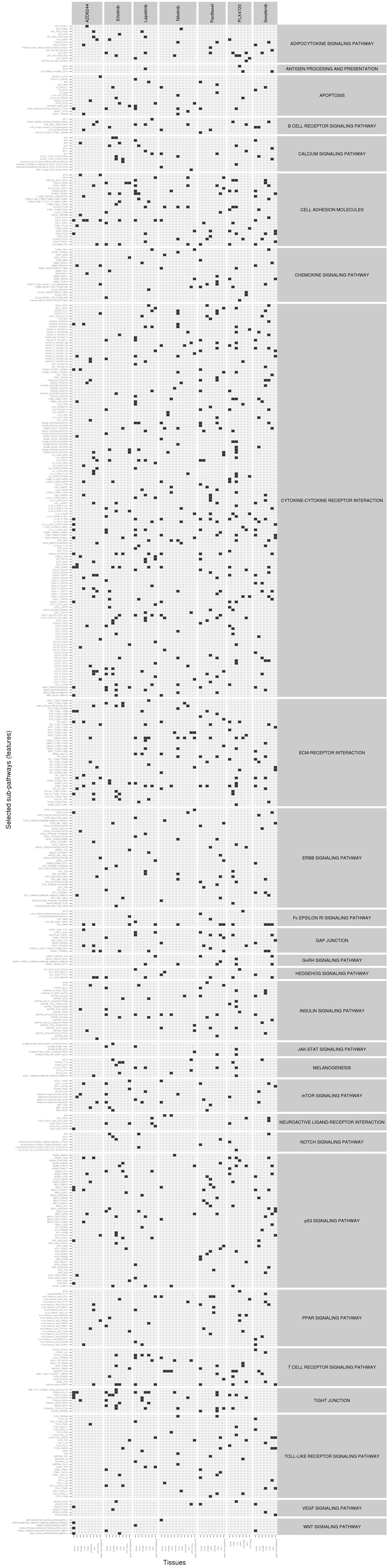
AZD6244
Erlotinib
Lapatinib
Nilotinib
Paclitaxel
PLX4720
Sorafenib
ADIPOCYTOKINE SIGNALING PATHWAY
ANTIGEN PROCESING AND PRESENTATION
APOPTOSIS
B CELL RECEPTOR SIGNALING PATHWAY
CALCIUM SIGNALING PATHWAY
CELL ADHESION MOLECULES
CHEMOKINE SIGNALING PATHWAY
CYTOKINE-CYTOKINE RECEPTOR INTERACTION
ECM-RECEPTOR INTERACTION
ERBB SIGNALING PATHWAY
Fc EPSILON RI SIGNALING PATHWAY
GAP JUNCTION
GnRH SIGNALING PATHWAY
HEDGEHOG SIGNALING PATHWAY
INSULIN SIGNALING PATHWAY
JAK-STAT SIGNALING PATHWAY
MELANOGENESIS
mTOR SIGNALING PATHWAY
NEUROACTIVE LIGAND-RECEPTOR INTERACTION
NOTCH SIGNALING PATHWAY
p53 SIGNALING PATHWAY
PPAR SIGNALING PATHWAY
T CELL RECEPTOR SIGNALING PATHWAY
TIGHT JUNCTION
TOLL-LIKE RECEPTOR SIGNALING PATHWAY
VEGF SIGNALING PATHWAY
WNT SIGNALING PATHWAY
Selected sub-pathways (features)
Tissues

Supplementary Table 1. Pathways in which the predictor selected signalling circuits with high predictive power and references that explain how the pathway is affected by the drug.

Compound	Pathway	References
**AZD6244**	Apoptosis	S. V. Holt et al., Enhanced apoptosis and tumor growth suppression elicited by combination of MEK (selumetinib) and mTOR kinase inhibitors (AZD8055). Cancer Res 72, 1804 (Apr 1, 2012).<br>S. Bhalla et al., The novel anti-MEK small molecule AZD6244 induces BIM-dependent and AKT-independent apoptosis in diffuse large B-cell lymphoma. Blood 118, 1052 (Jul 28, 2011).
	Insulin signaling pathway	Y. N. Gopal et al., Basal and treatment-induced activation of AKT mediates resistance to cell death by AZD6244 (ARRY-142886) in Braf-mutant human cutaneous melanoma cells. Cancer Res 70, 8736 (Nov 1, 2010).
	mTOR signaling pathway	H. Huynh, AZD6244 (ARRY-142886) enhances the antitumor activity of rapamycin in mouse models of human hepatocellular carcinoma. Cancer 116, 1315 (Mar 1, 2010).
	WNT signaling pathway	J. J. Tentler et al., Identification of predictive markers of response to the MEK1/2 inhibitor selumetinib (AZD6244) in K-ras-mutated colorectal cancer. Mol Cancer Ther 9, 3351 (Dec, 2010).
**Erlotinib**	Adipocytokine signaling pathway	N. K. Saxena et al., Bidirectional crosstalk between leptin and insulin-like growth factor-I signaling promotes invasion and migration of breast cancer cells via transactivation of epidermal growth factor receptor. Cancer Res 68, 9712 (Dec 1, 2008).
	Calcium signaling pathway	J. A. Bryant, R. S. Finn, D. J. Slamon, T. F. Cloughesy, A. C. Charles, EGF activates intracellular and intercellular calcium signaling by distinct pathways in tumor cells. Cancer Biol Ther 3, 1243 (Dec, 2004).
	ERBB signaling pathway	R. Roskoski, Jr., The ErbB/HER family of protein-tyrosine kinases and cancer. Pharmacol Res 79, 34 (Jan, 2014).
	Hedgehog signaling pathway	A. Ahmad et al., Inhibition of Hedgehog signaling sensitizes NSCLC cells to standard therapies through modulation of EMT-regulating miRNAs. Journal of hematology & oncology 6, 77 (2013).
	JAK-STAT signaling pathway	P. Uribe, S. Gonzalez, Epidermal growth factor receptor (EGFR) and squamous cell carcinoma of the skin: molecular bases for EGFR-targeted therapy. Pathology, research and practice 207, 337 (Jun 15, 2011).

	mTOR signaling pathway	J. Bliesath et al., Combined inhibition of EGFR and CK2 augments the attenuation of PI3K-Akt-mTOR signaling and the killing of cancer cells. Cancer Lett 322, 113 (Sep 1, 2012).
	p53 signaling pathway	A. R. Amin, F. R. Khuri, Z. G. Chen, D. M. Shin, Synergistic growth inhibition of squamous cell carcinoma of the head and neck by erlotinib and epigallocatechin-3-gallate: the role of p53-dependent inhibition of nuclear factor-kappaB. Cancer Prev Res (Phila) 2, 538 (Jun, 2009).
	WNT signaling pathway	M. Casas-Selves et al., Tankyrase and the canonical Wnt pathway protect lung cancer cells from EGFR inhibition. Cancer Res 72, 4154 (Aug 15, 2012).
**Lapatinib**	Adipocytokine signaling pathway	N. K. Saxena et al., Bidirectional crosstalk between leptin and insulin-like growth factor-I signaling promotes invasion and migration of breast cancer cells via transactivation of epidermal growth factor receptor. Cancer Res 68, 9712 (Dec 1, 2008).
	Apoptosis	X. H. Long et al., Lapatinib alters the malignant phenotype of osteosarcoma cells via downregulation of the activity of the HER2-PI3K/AKT-FASN axis in vitro. Oncol Rep 31, 328 (Jan, 2014).<br>H. L. Huang et al., Lapatinib induces autophagy, apoptosis and megakaryocytic differentiation in chronic myelogenous leukemia K562 cells. PLoS ONE 6, e29014 (2011).<br>A. Singh et al., Profiling pathway-specific novel therapeutics in preclinical assessment for central nervous system atypical teratoid rhabdoid tumors (CNS ATRT): favorable activity of targeting EGFR- ErbB2 signaling with lapatinib. Mol Oncol 7, 497 (Jun, 2013).
	ERBB signaling pathway	R. Roskoski, Jr., The ErbB/HER family of protein-tyrosine kinases and cancer. Pharmacol Res 79, 34 (Jan, 2014).
	Insulin signaling pathway	N. A. O'Brien et al., Activated phosphoinositide 3-kinase/AKT signaling confers resistance to trastuzumab but not lapatinib. Mol Cancer Ther 9, 1489 (Jun, 2010).
	mTOR signaling pathway	X. Zhu et al., Autophagy stimulates apoptosis in HER2-overexpressing breast cancers treated by lapatinib. J Cell Biochem 114, 2643 (Dec, 2013).
**Nilotinib**	Apoptosis	M. E. Shaker, A. Ghani, G. E. Shiha, T. M. Ibrahim, W. Z. Mehal, Nilotinib induces apoptosis and autophagic cell death of activated hepatic stellate cells via inhibition of histone deacetylases. Biochim Biophys Acta 1833, 1992 (Aug, 2013).<br>20. L. M. Packer et al., Nilotinib and MEK inhibitors induce synthetic lethality through paradoxical activation of RAF in drug-resistant chronic myeloid leukemia. Cancer Cell 20, 715 (Dec 13, 2011).
	Hedgehog signaling pathway	M. Cea et al., Tracking molecular relapse of chronic myeloid leukemia by

		measuring Hedgehog signaling status. Leuk Lymphoma 54, 342 (Feb, 2013).
**Paclitaxel**	Apoptosis	S. T. Park et al., Tumor suppressor BLU promotes paclitaxel antitumor activity by inducing apoptosis through the down-regulation of Bcl-2 expression in tumorigenesis. Biochem Biophys Res Commun 435, 153 (May 24, 2013).<br>Y. Wu et al., A combination of paclitaxel and siRNA-mediated silencing of Stathmin inhibits growth and promotes apoptosis of nasopharyngeal carcinoma cells. Cell Oncol (Dordr) 37, 53 (Feb, 2014).<br>A. V. Miller et al., Paclitaxel-induced apoptosis is BAK-dependent, but BAX and BIM-independent in breast tumor. PLoS ONE 8, e60685 (2013).
	Calcium signaling pathway	W. Boehmerle et al., Paclitaxel induces calcium oscillations via an inositol 1,4,5-trisphosphate receptor and neuronal calcium sensor 1-dependent mechanism. Proc Natl Acad Sci U S A 103, 18356 (Nov 28, 2006).
	Chemokine signaling pathway	R. W. Jackman, M. G. Rhoads, E. Cornwell, S. C. Kandarian, Microtubule-mediated NF-kappaB activation in the TNF-alpha signaling pathway. Exp Cell Res 315, 3242 (Nov 15, 2009).
	Hedgehog signaling pathway	H. Tanaka et al., The Hedgehog signaling pathway plays an essential role in maintaining the CD44+CD24-/low subpopulation and the side population of breast cancer cells. Anticancer Res 29, 2147 (Jun, 2009).
	Insulin signaling pathway	R. Dziadziuszko, D. R. Camidge, F. R. Hirsch, The insulin-like growth factor pathway in lung cancer. J Thorac Oncol 3, 815 (Aug, 2008).
	JAK-STAT signaling pathway	T. H. Wang et al., Paclitaxel (Taxol) upregulates expression of functional interleukin-6 in human ovarian cancer cells through multiple signaling pathways. Oncogene 25, 4857 (Aug 10, 2006).
	p53 signaling pathway	E. Pasquier et al., Antiangiogenic activity of paclitaxel is associated with its cytostatic effect, mediated by the initiation but not completion of a mitochondrial apoptotic signaling pathway. Mol Cancer Ther 3, 1301 (Oct, 2004).
	PPAR signaling pathway	Y. Z. Chen et al., PPAR signaling pathway may be an important predictor of breast cancer response to neoadjuvant chemotherapy. Cancer Chemother Pharmacol 70, 637 (Nov, 2012).
	Toll-like receptor signaling pathway	T. H. Wang et al., Paclitaxel (Taxol) upregulates expression of functional interleukin-6 in human ovarian cancer cells through multiple signaling pathways. Oncogene 25, 4857 (Aug 10, 2006).
	VEGF signaling pathway	H. Cheng et al., In vitro sequence-dependent synergism between paclitaxel and gefitinib in human lung cancer cell lines. Cancer Chemother Pharmacol 67, 637 (Mar, 2011).
	WNT signaling pathway	A. M. Sanchez et al., Treatment with anticancer agents induces dysregulation of

		specific Wnt signaling pathways in human ovarian luteinized granulosa cells in vitro. Toxicol Sci 136, 183 (Nov, 2013).
**PLX4720**	Apoptosis	F. M. Kaplan, Y. Shao, M. M. Mayberry, A. E. Aplin, Hyperactivation of MEK-ERK1/2 signaling and resistance to apoptosis induced by the oncogenic B-RAF inhibitor, PLX4720, in mutant N-RAS melanoma cells. Oncogene 30, 366 (Jan 20, 2011).<br>C. C. Jiang et al., MEK-independent survival of B-RAFV600E melanoma cells selected for resistance to apoptosis induced by the RAF inhibitor PLX4720. Clin Cancer Res 17, 721 (Feb 15, 2011).
	WNT signaling pathway	T. L. Biechele et al., Wnt/beta-catenin signaling and AXIN1 regulate apoptosis triggered by inhibition of the mutant kinase BRAFV600E in human melanoma. Sci Signal 5, ra3 (Jan 10, 2012).
**Sorafenib**	Apoptosis	N. K. Sun, S. L. Huang, T. C. Chang, C. C. Chao, Sorafenib induces endometrial carcinoma apoptosis by inhibiting Elk-1-dependent Mcl-1 transcription and inducing Akt/GSK3beta-dependent protein degradation. J Cell Biochem 114, 1819 (Aug, 2013).<br>X. F. Li et al., Sorafenib down-regulates c-IAP expression post-transcriptionally in hepatic carcinoma cells to suppress apoptosis. Biochem Biophys Res Commun 418, 531 (Feb 17, 2012).
	B cell receptor signaling pathway	P. Robak, T. Robak, A targeted therapy for protein and lipid kinases in chronic lymphocytic leukemia. Current medicinal chemistry 19, 5294 (2012).
	Chemokine signaling pathway	M. Gassenmaier et al., CXC chemokine receptor 4 is essential for maintenance of renal cell carcinoma-initiating cells and predicts metastasis. Stem Cells 31, 1467 (Aug, 2013).
	ERBB signaling pathway	A. Sartore-Bianchi, R. Ricotta, G. Cerea, M. R. Maugeri, S. Siena, Rationale and clinical results of multi-target treatments in oncology. The International journal of biological markers 22, S77 (Jan-Mar, 2007).
	Hedgehog signaling pathway	A. Moeini, H. Cornella, A. Villanueva, Emerging signaling pathways in hepatocellular carcinoma. Liver cancer 1, 83 (Sep, 2012).
	Insulin signaling pathway	H. Huynh, AZD6244 (ARRY-142886) enhances the antitumor activity of rapamycin in mouse models of human hepatocellular carcinoma. Cancer 116, 1315 (Mar 1, 2010).<br>D. L. Ou et al., Potentiating the efficacy of molecular targeted therapy for hepatocellular carcinoma by inhibiting the insulin-like growth factor pathway. PLoS ONE 8, e66589 (2013).
	JAK-STAT signaling pathway	B. R. Blechacz et al., Sorafenib inhibits signal transducer and activator of

	transcription-3 signaling in cholangiocarcinoma cells by activating the phosphatase shatterproof 2. Hepatology 50, 1861 (Dec, 2009).
mTOR signaling pathway	H. Huynh, AZD6244 (ARRY-142886) enhances the antitumor activity of rapamycin in mouse models of human hepatocellular carcinoma. Cancer 116, 1315 (Mar 1, 2010).<br>R. Gedaly et al., PI-103 and sorafenib inhibit hepatocellular carcinoma cell proliferation by blocking Ras/Raf/MAPK and PI3K/AKT/mTOR pathways. Anticancer Res 30, 4951 (Dec, 2010).
p53 signaling pathway	C. Dudgeon et al., Inhibiting oncogenic signaling by sorafenib activates PUMA via GSK3beta and NF-kappaB to suppress tumor cell growth. Oncogene 31, 4848 (Nov 15, 2012).
VEGF signaling pathway	S. Shimizu et al., Inhibition of autophagy potentiates the antitumor effect of the multikinase inhibitor sorafenib in hepatocellular carcinoma. Int J Cancer 131, 548 (Aug 1, 2012).
WNT signaling pathway	A. Lachenmayer et al., Wnt-pathway activation in two molecular classes of hepatocellular carcinoma and experimental modulation by sorafenib. Clin Cancer Res 18, 4997 (Sep 15, 2012).
